# Azathioprine Hypersensitivity Syndrome during Treatment of Severe Interstitial Lung Disease with Anti-Neutrophil Cytoplasmic Antibody-Associated Vasculitis

**DOI:** 10.1155/2020/8852441

**Published:** 2020-07-03

**Authors:** Eri Nakano, Tomohiko Asakawa, Mea Asou, Eri Nohara, Tomoyuki Seki, Makoto Araki

**Affiliations:** ^1^Department of Internal Medicine, Suwa Central Hospital, Chino, Nagano 391-8503, Japan; ^2^National Hospital Organization, Tokyo National Hospital, Takeoka, Kiyose, Tokyo 204-8585, Japan

## Abstract

Azathioprine is used to treat anti-neutrophil cytoplasmic antibody- (ANCA-) associated vasculitis. Azathioprine hypersensitivity syndrome is often missed. An 81-year-old man undergoing treatment for interstitial pneumonia developed a high fever and was diagnosed with ANCA-associated vasculitis based on an elevated myeloperoxidase- (MPO-) ANCA titer and renal biopsy findings. After induction therapy, his clinical symptoms improved, but his MPO-ANCA remained elevated (>300 U·L^−1^) and hematuria persisted. Prednisolone plus azathioprine was administered as maintenance therapy. Three exacerbations of the inflammatory response occurred during the subsequent 3 months. In each instance, we suspected opportunistic infection or a flare-up of vasculitis. The first exacerbation was treated with an increased prednisolone dose and antibiotics. At the onset of the second exacerbation, which was accompanied by systemic erythema, we stopped azathioprine and administered antibiotics. The third exacerbation, which occurred the day after restarting azathioprine, involved a fever with chills and an acute inflammatory reaction; we therefore suspected an azathioprine allergy. A drug provocation test was performed, and a hyperinflammatory response was observed. The patient received prednisolone (15 mg·day^−1^) monotherapy; no further fever was observed during the subsequent 2 months. We therefore diagnosed azathioprine hypersensitivity syndrome. Under treatment with prednisolone (5 mg·day^−1^) and mycophenolate mofetil (1 g·day^−1^) (replacing the azathioprine), no signs of relapse or infection have occurred for more than two years. Renal function and the pulmonary lesions are stable, although the high MPO-ANCA titer and hematuria persist. The diagnosis of azathioprine hypersensitivity is often delayed because of the difficulty in identifying the relationship between immunosuppressive agents and hypersensitivity and in distinguishing this from infection or relapse of the primary disease. The misdiagnosis of azathioprine hypersensitivity leads to unnecessary treatment; thus, clinicians should consider allergic reactions specific to azathioprine when switching from induction to maintenance therapy.

## 1. Introduction

In the treatment of anti-neutrophil cytoplasmic antibody- (ANCA-) associated vasculitis (AAV), powerful immunosuppressive drugs are usually used, which can lead to treatment-related deaths from infection. Therefore, management of vasculitis flare-ups and opportunistic infections is always important in AAV. Azathioprine (AZA) is the standard AAV maintenance therapy. AZA hypersensitivity syndrome was previously considered to be a rare side effect of AZA, but a recent report found that it occurs in 9% of AZA patients [1]. This syndrome is often misdiagnosed as infection or disease exacerbation and thus is prone to mistreatment; a thorough understanding of its clinical manifestations is therefore required. We report a case of AZA hypersensitivity syndrome that occurred during the treatment of severe interstitial lung disease with AAV.

## 2. Case Presentation

An 81-year-old man with no history of smoking had been undergoing treatment for chronic interstitial pneumonia and paroxysmal atrial fibrillation for 6 years. Incomplete investigations had failed to identify the cause of the interstitial pneumonia. A high fever, worsening of renal function, and myeloperoxidase- (MPO-) ANCA positivity had been noted 6 months earlier (Table 1). A renal biopsy revealed global sclerosis in 2 of 12 glomeruli and 3 glomeruli with glomerular basement membrane necrosis. Crescent formation was not observed. AAV was diagnosed based on the pauci-immune pattern of immunofluorescent staining. As the patient had a resolved hepatitis B virus (HBV) infection (negative for Hbs antigen and positive for Hbc antibody), we decided to perform the standard recommended induction therapy, with prednisolone (PSL) and six courses of intravenous cyclophosphamide (CYC) [2].

After induction therapy, the inflammatory response promptly improved. Renal function remained almost unchanged, but hematuria persisted. We decided to ignore this, considering the impact of direct oral anticoagulants. Lung function did not return, and home oxygen therapy was introduced. The ANCA titer remained over 300 U·mL^−1^. As there was no change in the Vasculitis Damage Index (VDI) (score of 5; pulmonary fibrosis, chronic breathlessness, impaired lung function, hypertension, and estimated glomerular filtration rate < 50 mL·min^−1^·1.73 m^(2)-1^), we considered organ failure and decided to shift from induction therapy to maintenance therapy. AZA was selected as an adjunctive immunosuppressive agent to the current PSL (10 mg·day^−1^) regimen. Owing to the potential for hepatic impairment due to AZA, we titrated AZA (25 mg·day^−1^) in hospital.

**Figure 1:**
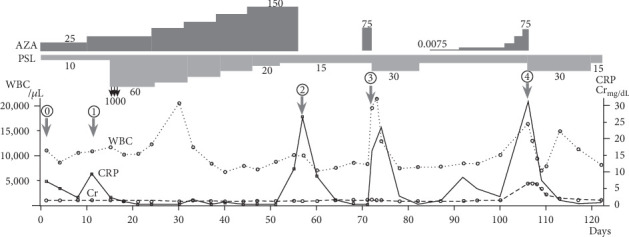
WBC, CRP, and Cr levels at about 4 months after the start of AZA and trends in PSL and AZA medication doses. Blood test results for the days indicated by the bold arrow are shown in Table 1. AZA: azathioprine; PSL: prednisolone.

**Figure 2:**
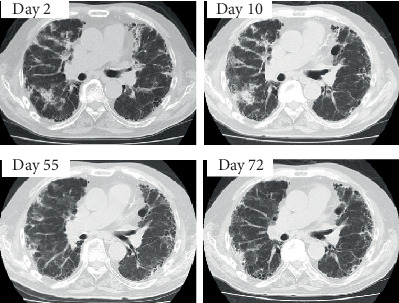
Changes in chest CT. Date is the number of days after starting AZA. Day 2: diffuse shading, and a regional infiltrative shadow in the right lower lobe is observed. Day 10: scattered nonregional increases in concentration are the same as before. No apparent exacerbation. Day 55: scattered irregular infiltrative shadows in both peripheral lungs and nondistrictal frosted concentrations are observed. Day 72: distinct infiltrative shadows scattered in the periphery of both lungs, with no significant changes.

After 10 days of AZA administration (Day 10), blood tests revealed a white blood cell (WBC) count of 10,840 *μ*L^−1^, a creatine (Cr) level of 1.55 mg·dL^−1^, and a C reactive protein (CRP) level of 9.23 mg·dL^−1^; a mild worsening of the inflammatory response and renal function was also observed (Figure 1, ①, Table 1, ①). The patient had no fever and stable breathing. A chest computed tomography (CT) scan was performed, which showed a worsening right lower lobe infiltration shadow, but could not distinguish between an exacerbation of interstitial pneumonia and infection (Figure 2). The high ANCA titers and persistent hematuria remained. Beta-D-glucan and cytomegalovirus- (CMV-) antigenemia tests were negative. There were no abnormal findings in the sputum culture, including mycobacteria. In the absence of clear findings, we decided to treat the infection and vasculitis. For the bacterial pneumonia, ceftriaxone 2 g·day^−1^ was administered for 2 weeks. For the vasculitis, high-dose methylprednisolone therapy (1,000 mg·day^−1^ for 3 days) was performed. Thereafter, the patient was phased down from 60 mg·day^−1^ PSL while AZA treatment was phased up.

**Figure 3:**
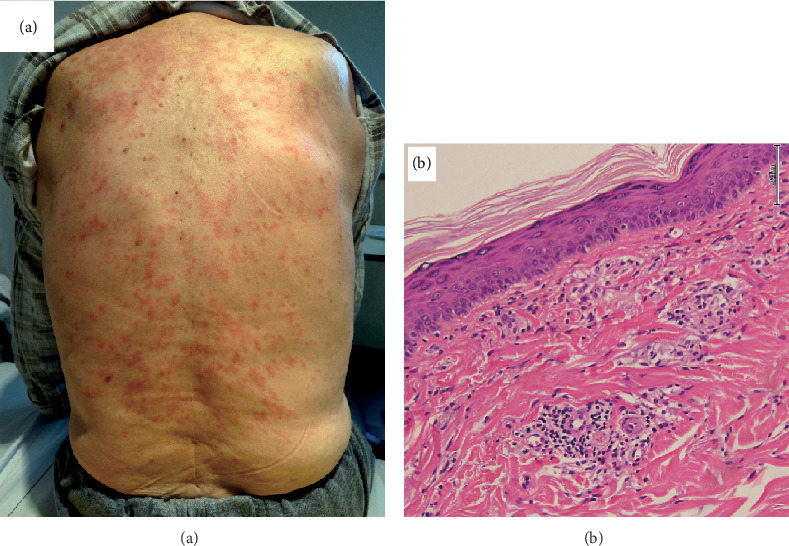
(a) There is a rash all over the body, mainly on the back. (b) Mild focal neutrophilic infiltration in the superficial dermis and mild to moderate perivascular lymphocytic infiltration. There are no vasculitis findings. Haematoxylin and eosin stain. 200x.

On Day 52, the patient was discharged with prescriptions for PSL (15 mg·day^−1^) and AZA (150 mg·day^−1^). However, 3 days later, the patient developed a fever of 40.3°C with chills and was urgently admitted to our hospital (Figure 1, ②). Systemic erythema was observed without mucosal damage (Figure 3(a)). A skin biopsy was performed, and the patient was diagnosed with erythema exudativum multiforme based on mild neutrophil infiltration in the shallow dermis (Figure 3(b)). However, we did not know whether this was caused by the drug or the infection. A CT scan was performed, but the invasive shadow of the right lower lobe had improved. Blood tests and imaging studies showed that neither pneumonia nor vasculitis was likely (Figure 2, Table 1, ②). As lymphocytopenia (444 *μ*L^−1^), probably owing to AZA, was observed, we considered this to be the cause of the infection, discontinued AZA, and commenced levofloxacin (500 mg·day^−1^) for 14 days. On Day 10 of hospitalization (Day 65), the CRP level was 1.54 mg·dL^−1^ and the inflammatory response had improved.

On Day 71, as his general condition improved, the patient resumed AZA (75 mg·day^−1^). The next day, at 15:00, the patient developed a fever of 39.3°C with shivering chills (Figure 1, ③). Although blood tests showed a WBC count of 8,330 *μ*L^−1^ and a CRP level of 0.17 mg·dL^−1^ on Day 71, these counts worsened, to a WBC count of 19,650 *μ*L^−1^ and a CRP level of 16.19 mg·dL^−1^ in less than 30 hours (Table 1, ③). We performed a systemic search, which included the respiratory (Figure 2) and urinary tracts, but neither test findings nor urine and blood cultures produced significant evidence of infection. The antibiotic combination of imipenem/cilastatin (0.5 g·12 h^−1^) was administered, AZA was discontinued, and the dose of PSL was transiently increased to 30 mg as the patient was considered to be under high stress.

As the disease worsened after the administration of AZA, we thought it was likely drug-related, although there was no eosinophilia. However, the high MPO-ANCA titer and microscopic hematuria findings did not rule out a relapse of AAV. In addition, there were few alternative drugs available to treat this patient owing to the severe lung damage and history of HBV infection. Therefore, approximately 2 weeks after the third fever (Day 85), we initiated a drug provocation test with the patient's consent. The dose of AZA was increased to 0.75, 1.5, 3, 6, 12.5, 25, and 50, then 75 mg·day^−1^ at intervals of 2 to 3 days. We monitored the patient's vital signs throughout the course, and the patient did not develop a fever or rash during the 3 weeks of escalation. Up to Day 16 of the drug provocation test (Day 100, AZA 12.5 mg·day^−1^), the WBC count (10,090 *μ*L^−1^), CRP level (2.56 mg·dL^−1^), and Cr level (1.49 mg·dL^−1^) were acceptable. On Day 106 (AZA 75 mg·day^−1^), there was no fever but the inflammatory response increased and renal function worsened, as the WBC count (16,410 *μ*L^−1^), CRP level (31.19 mg·dL^−1^), and Cr level (6.4 mg·dL^−1^) increased (Figure 1, ④, Table 1, ④). AZA was immediately discontinued, and the dose of PSL was doubled to 30 mg·day^−1^. No antibiotics were used. Renal function returned, indicated by a drop in the Cr level from 6.4 to 1.5 mg·dL^−1^ after 11 days, and the inflammatory response improved. We decided to confirm that the disease would not recur in the absence of AZA. The patient received PSL (15 mg) monotherapy for 2 months without the occurrence of fever or exacerbated inflammatory response; renal function did not worsen. The patient was therefore diagnosed with AZA hypersensitivity syndrome.

Mycophenolate mofetil (MMF) was selected as an additional immunosuppressive agent. The dose of PSL was reduced, and MMF was introduced 2 months after the discontinuation of AZA. Taking into account renal function and old age, the dose of MMF was set at 1 g. For more than 2 years, PSL (5 mg·day^−1^) and MMF (1 g·day^−1^) have been administered and there was no fever or acute inflammatory reaction. The patient's breathing and renal function have stabilized. The high MPO-ANCA titer and urine occult blood findings remain unchanged.

## 3. Discussion

AZA hypersensitivity is a relatively common condition that requires a change in treatment and should not be overlooked. However, in practice, the diagnosis is often difficult because the syndrome mimics infection or the recurrence of AAV. Infection is the main cause of death in AAV, and, in a case of severe pulmonary impairment with a poor reserve capacity, such as this one, the possibility of respiratory infection during a hyperinflammatory response must always be considered. In this case, the MPO-ANCA was persistently >300 U·L^−1^ and urine occult blood was >50 red blood cells per high-power field; thus, the possibility of AAV relapse should be considered. We think that it is meaningful to report how we responded to the allergic reaction to this immunosuppressive drug in such a situation.

AZA hypersensitivity syndrome, a recently proposed concept, is a febrile rash disease that appears, on average, 13.3 days (5–28 days) after the initiation of AZA [3]. This syndrome used to be considered rare, but recently, it has been reported to occur in 9% of patients receiving AZA; thus, it is an important side effect to be aware of [1]. Background diseases, such as AAV, Crohn's disease, ulcerative colitis, autoimmune hepatitis, and chronic rheumatoid arthritis, have been reported with AZA hypersensitivity syndrome, but there is no significant trend. Overall, 49% of AZA hypersensitivity syndromes present with cutaneous symptoms, and the typical rash is Sweet's syndrome-like neutrophilic dermatosis [4]. Symptoms other than fever and rash, such as nausea, vomiting, and meningitis, have also been reported [5]. However, as none are specific to AZA hypersensitivity, it is often overlooked, the symptoms being considered to be dermatosis associated with the underlying disease or infection [6].

In this case, we had difficulty diagnosing AZA hypersensitivity syndrome. In general, most fevers during collagen disease therapy are aggravations of the underlying disease or infections [7], so drug allergies are unlikely to be considered. Moreover, although drug fever has been reported not only with AZA but also with other immunosuppressive agents, such as CYC, mizoribine, and MMF [8–10], allergic reactions to immunosuppressive agents are difficult to recognize in a patient. In the present case, the worsening of the inflammatory response on Day 10 was considered to be either infection or exacerbation of AAV, not an allergic reaction to AZA. The reasons were that the fever was mild, there was no rash, high MPO-ANCA titer and hematuria persisted, and the CT scan showed an infiltrative shadow in the right lower lobe. Furthermore, the acute inflammatory reaction on Day 55 should be diagnosed as AZA hypersensitivity because there was no change in imaging findings and a typical skin rash appeared. However, we did not consider it to be AZA-induced hypersensitivity, as most hypersensitivity cases occur within the first month of treatment. We also did not find that the hypersensitivity was suppressed by high doses of prednisolone.

AZA hypersensitivity syndrome is thought to be mainly caused by the accumulation of toxic metabolites in conjunction with a decrease in thiopurine methyltransferase (TPMT) activity [1]. Unfortunately, TPMT activity could not be measured in this case. Currently, there is no reliable skin test or *in vitro* study for the diagnosis of AZA hypersensitivity. As we did not want to use rituximab (RTX) or methotrexate (MTX) because of the patient's history of the patient's background, such as a history of hepatitis B infection or pulmonary fibrosis, we decided to confirm the diagnosis of AZA using a drug provocation test. We titrated a small dose of AZA, which was the same drug he used at home, and administered increasing doses (Figure 1, PSL dose is fixed at 15 mg·day^−1^), as performed previously [11]. In this test, the absence of a response at low doses of AZA and the onset of AZA hypersensitivity syndrome at higher doses probably represented dose dependency, as the cause of AZA hypersensitivity is thought to be a decrease in the activity of TPMT. Allergy to AZA was further demonstrated by the absence of inflammatory reactions in the reverse test (2 months of PSL monotherapy).

A similar case of renal impairment owing to AZA hypersensitivity syndrome has been reported in which the Cr level rose on Day 10 from 1.5 to 5.2 mg·dL^−1^ after the administration of 150 mg of AZA. When AZA was resumed after 9 months of CYC treatment, fever and acute renal failure, with a Cr level of 3.0 mg·dL^−1^, developed within a few hours owing to suspected recurrence of AZA hypersensitivity [12]. Interstitial damage has been suggested as a mechanism for this renal impairment [12, 13].

Remission is defined as the complete absence of clinical disease activity [14]; however, it is difficult to assess when severe respiratory failure occurs, such as in this case. This case switched from induction therapy to maintenance therapy because the VDI score did not worsen. However, it was unclear whether the kidneys were in remission because the urine occult blood (occasionally with dysmorphic erythrocytes) was always positive, partly owing to the antithrombotic therapy. Furthermore, the MPO-ANCA was always high in this case. There is currently a debate about whether MPO-ANCA predicts the future recurrence of AAV, but it is generally not recommended as an indicator, and the risk of recurrence must be judged in relation to the general condition of the patient [2]. However, it has been reported that a positive ANCA at the start of maintenance therapy is associated with a higher risk of AAV recurrence [15], which could not be ignored in this case. It is unclear why ANCA was always high in this case, but the clinical course suggests that there was no morbidity. According to a report, 1 in 56 patients with AAV had a consistently high MPO-ANCA level but did not relapse [16]. There is also a report that MPO-ANCA patients with persistently elevated MPO-ANCA levels do not relapse but do develop chronic renal failure [17]. Additionally, a study has shown that differences in the epitopes of ANCA determine whether they are pathogenic [18].

When switching to maintenance therapy, AAV should be treated with a combination of low-dose PSL and an immunosuppressive agent. The European League Against Rheumatism recommends, in order of priority, AZA, RTX, MTX, and MMF as maintenance therapy and considers leflunomide (LEF) when these are not available. RTX has the serious side effects of opportunistic infection and viral reactivation. When RTX is used to treat lymphoma, HBV reactivation is observed in 8% of patients with HBsAg-negative and anti-HBc-positive HBV [19]. In this case, the HBV-DNA level was below the measurement sensitivity level, and, although it was possible to monitor the HBV-DNA level periodically, we decided not to because of the risk of opportunistic infection. MTX and LEF could not be used in this case of severe lung damage because of interstitial pneumonia. AZA is recommended over MMF for the treatment of AAV because patients treated with MMF had a higher relapse rate than those treated with AZA in the IMPROVE trial [20]. Additionally, the side effects of MMF include the risk of viral infections, gastrointestinal symptoms, myelosuppression, and CMV infection. However, MMF causes fewer adverse events in the lungs than MTX. Therefore, we decided to use MMF in this case, considering the patient's renal function. Although there is no established recommendation for the duration of remission maintenance therapy for AAV, it is desirable to continue for at least 24 months after the induction of maintenance therapy.

## 4. Conclusion

AZA hypersensitivity syndrome is a Sweet's syndrome-like febrile rash that occurs within 4 weeks of AZA initiation. Infection and flare-ups are a concern in the treatment of AAV, which is heightened if AAV is accompanied by severe lung damage. In such circumstances, immunosuppressive drug allergies can go unnoticed and are prone to misdiagnosis and overtreatment. Clinicians should be aware of this case and consider the possibility of allergic reactions specific to AZA when switching from induction therapy to maintenance therapy.

## Figures and Tables

**Table 1:** Laboratory findings during the course of the disease.

	At diagnosis	⓪	①	②	③	④	2 years after MMF therapy	Units
WBC	12,570	10,990	10,840	10,100	19,650	16,410	6,030	/*μ*L
Hb	12.2	12.1	12.1	12.3	14	11.8	10.4	mg/dL
Plt	43.7	35.8	32.8	19.4	52.6	26.7	34.0	10^4^/*μ*L
Alb	2.5	3.1	3.1		3.0	2.4	3.8	mg/dL
LDH	209	264	232	300	244	334	206	IU/L
AST	29	18	36	24	35	50	22	IU/L
ALT	37	15	30	26	28	39	10	IU/L
CPK	54	19	17	38	20	38	43	IU/L
BUN	20.9	26.1	28.5	20.8	34.1	70.4	18.2	mg/dL
Cr	0.93	1.40	1.55	1.30	1.66	6.34	1.24	mg/dL
Na	137	140	138	139	137	136	141	mEq/L
K	4.6	4.6	5.5	4.2	4.3	4.9	4.0	mEq/L
Cl	104	104	102	103	101	103	100	mEq/L
CRP	19.92	2.22	9.23	10.87	16.19	31.19	0.43	mg/dL
Urine protein	2+	−	−	2+	±	2+	−	
Urine occult blood	3+	3+	3+	3+	3+	3+	2+	
Urine sediment (RBC)	50-99	50-99	50-99	>100	>100	>100	50-99	/HPF
*β*-D-Glucan		9.6		11.9	7.7			pg/mL
CMV-antigenemia		neg.		neg.	neg.			
SP-D				<17.2	23.7			mg/dL
KL-6					1,220		469	U/mL
IgG		731		583				mg/dL
Anti-MPO-ANCA	>300	>300		>300		>300	>300	U/mL
Anti-GBM-Ab	<2.0					<2.0		U/mL
Anti-PR3-ANCA	<1.0							U/mL
Anti-nuclear antibody	×40							

ANCA: anti-neutrophil cytoplasmic antibodies; CMV: cytomegalovirus; GBM: glomerular basement membrane; KL-6: Krebs von den Lungen-6; MMF: mycophenolate mofetil; MPO: myeloperoxidase; PR3: proteinase 3; SP-D: surfactant protein d; RBC: red blood cell.

## Data Availability

No data were used to support this study.
